# Effect of orthodontic forces on levels of enzymes in gingival crevicular fluid (GCF): A systematic review

**DOI:** 10.1590/2177-6709.24.2.40.e1-22.onl

**Published:** 2019

**Authors:** Priyanka Kapoor, Nitika Monga, Om Prakash Kharbanda, Sunil Kapila, Ragini Miglani, Rajeswari Moganty

**Affiliations:** 1 Jamia Millia Islamia, Faculty of Dentistry, Department of Orthodontics (New Delhi, India).; 2 All India Institute of Medical Sciences, Centre for Dental Education and Research, Division of Orthodontics and Dentofacial Deformities (New Delhi, India).; 3 University of California San Francisco, Division of Orthodontics (San Francisco/CA, USA).; 4 All India Institute of Medical Sciences,Department of Biochemistry (New Delhi, India).

**Keywords:** Tooth movement, Gingival crevicular fluid (GCF), Enzymes, Systematic review

## Abstract

**Objective:**

Orthodontic force application releases multiple enzymes in gingival crevicular fluid (GCF) for activation, resorption, reversal, deposition of osseous elements and extracellular matrix degradation. The current systematic review critically evaluated all existing evidence on enzymes in orthodontic tooth movement.

**Methods:**

Literature was searched with predetermined search strategy on electronic databases (PubMed, Scopus, Embase), along with hand search.

**Results:**

Initial search identified 652 studies, shortlisted to 52 studies based on PRISMA. Quality assessment further led to final inclusion of 48 studies (13 moderately and 35 highly sensitive studies). Primary outcomes are significant upregulation in GCF levels of enzymes-aspartate aminotransferase (AST), alkaline phosphatase (ALP), matrix metalloproteinases (MMPs), lactate dehydrogenase (LDH), β-glucuronidase (βG), tartrate resistant acid phosphatase (TRAP), acid phosphatase (ACP) and down regulation in cathepsin B (Cb). Site specificity is shown by ALP, TRAP, AST, LDH, MMP9 with levels at compression site increasing earlier and in higher quantities compared with tension site. ALP levels are higher at tension site only in retention. A positive correlation of LDH, ALP and AST is also observed with increasing orthodontic force magnitude.

**Conclusions:**

A strong evidence of variation in enzymes (ALP, AST, ACP TRAP, LDH, MMPs, Cb) in GCF is found in association with different magnitude, stages and sites of orthodontic force application.

## Resumo

**Objetivo:**

a aplicação da força ortodôntica libera múltiplas enzimas no fluído crevicular gengival (FCG), desencadeando a ativação, reabsorção, reversão, deposição de elementos ósseos e degradação da matriz extracelular. A presente revisão sistemática avaliou criticamente toda a evidência disponível sobre os níveis de enzimas durante a movimentação ortodôntica.

**Métodos:**

utilizando-se estratégias predeterminadas, foram realizadas buscas em bases de dados eletrônicas (PubMed, Scopus, Embase), sendo também feitas buscas manuais.

**Resultados:**

a busca inicial identificou 652 estudos e, com base nas diretrizes do PRISMA, foram selecionados 52 estudos. A avaliação qualitativa resultou na inclusão final de 48 estudos (13 estudos com moderada sensibilidade e 35 com alto nível de sensibilidade). Os desfechos primários foram o aumento significativo dos níveis no FCG das enzimas aspartato aminotransferase (AST), fosfatase alcalina (FA), metaloproteinases de matriz (MMPs), lactato desidrogenase (LDH), β-glucuronidase (βG), fosfatase ácido-resistente ao tartarato (TRAP), fosfatase ácida (FAC) e baixa regulação de catepsina B (Cb). Especificidade quanto ao local foi mostrada para FA, TRAP, AST, LDH e MMP9 com os níveis no lado de compressão aumentando mais rápido e em maiores quantidades, quando comparado ao lado de tensão. Os níveis de FA foram maiores no lado de tensão somente no período de contenção. Uma correlação positiva de LDH, FA e AST também foi observada à medida que a magnitude de força ortodôntica aumentou.

**Conclusões:**

há fortes evidências indicando que as variações nas enzimas (FA, AST, FAC, TRAP, LDH, MMPs, Cb) presentes no FCG estão associadas a diferentes magnitudes, estágios e locais de aplicação da força ortodôntica.

## INTRODUCTION

Orthodontic forces cause an initial inflammatory response followed by alterations in the vascular and neural envelope and perpetual bone and tissue remodelling accompanied by paracrine release of bioactive mediators.^1^ ^-^ ^3^ During orthodontic tooth movement (OTM), host-derived enzymes are released at various stages of activation, resorption, reversal and deposition of osseous elements and degradation of the extracellular matrix.^4^ Some of these enzymes have been identified in the periodontal (pdl) tissue of orthodontically moved teeth.^5^ Gingival crevicular fluid (GCF) is however a better choice for assessing biomolecules or mediators as sample collection is simple, sensitive, convenient, repetitive and non-invasive.^6^ Thus, the quantitative estimations of mediators in GCF reflect biochemical mechanisms associated with OTM. A systematic review (SR) by Kapoor et al^6^ in 2014 studied variation in GCF level of cytokines with type and magnitude of orthodontic forces and growth status of patients. It established a positive correlation of GCF activity index IL1RA (interleukin receptor antagonist)/ IL-1β) with intensity of pain and velocity of OTM and a negative correlation with growth status of patients. Besides cytokines, numerous other mediators also alter GCF during OTM, comprehensively reviewed in SR by Alhadlaq^3^ in 2015. This SR highlighted working mechanisms of multiple mediators but heterogeneity of studies precluded attainment of concrete conclusions. Hence, the present SR aims to assess only a single family of mediators, enzymes, to establish their clinical correlations on sequential release in different phases of OTM and varying magnitude of orthodontic forces.

Soluble enzymes like lactate dehydrogenase (LDH) and aspartate aminotransferase (AST) present in cytoplasm are known to release in GCF only after cellular necrosis or hyalinization with heavy orthodontic forces.^4^ Tartrate-resistant acid phosphatase (TRAP) and alkaline phosphatase (ALP) exhibit osteoclastic and osteoblastic activity, respectively,^1^ and are identified in areas of tension (TS) or compression (CS) of teeth undergoing OTM. Heavy orthopedic forces of rapid maxillary expansion show an increase of ß-glucuronidase (ßG) lysosomal enzyme upon release from polymorphonuclear (PMN) leukocytes.^7^ Rise in PMN granules in surrounding tissues after fixed orthodontic appliance activation also show increase in myeloperoxidase (MPO) 2 hours (hr) after activation, traced both in GCF and saliva.^8^

The evidence on enzymes in OTM is plenty but scattered and lacks critical appraisal. Hence, the current SR is conducted to establish associations of enzymes in GCF to the site of application, magnitude and type of force, patient’s growth status and the type of archwire ligation. 

## MATERIAL AND METHODS 

### Protocol and registration 

**Figure 1:**
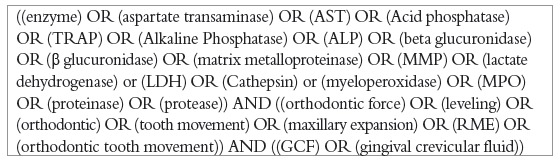
Search strategy applied on databases for inclusion of studies in the review.

**Figure 2:**
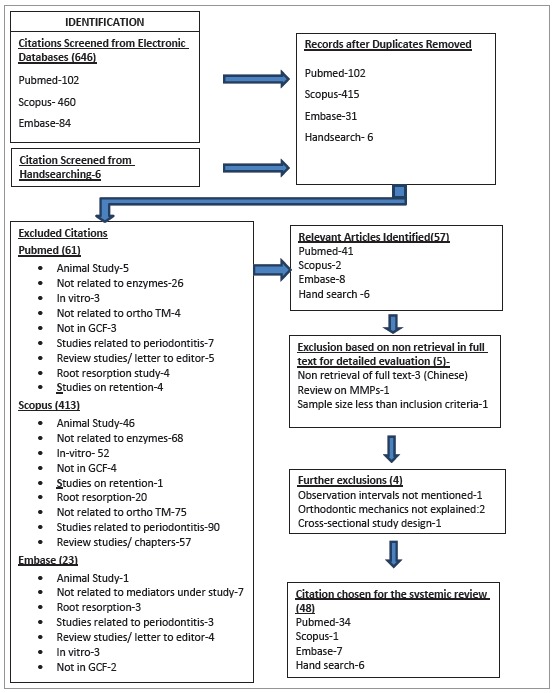
PRISMA flow diagram for inclusion of studies in the systematic review

The protocol for SR was registered in PROSPERO (www.crd.york.ac.uk/prospero, CRD42015017496) with a predetermined search strategy (Fig 1). It comprised of MeSH terms, Boolean terminology and free text terms with the keywords "enzyme" "protease", "orthodontic tooth movement" and "gingival crevicular fluid", together with several key enzymes. This search strategy was applied to key databases PubMed, Scopus and Embase in February 2018 with no language restrictions. Additional publications were identified through reference tracking and hand search of journals (Sains Malaysiana, Orthodontic Waves, Journal of Applied Sciences, APMC). The search was performed by two reviewers, followed by a cross-check by a third reviewer, in conformity with PRISMA, as shown in Figure 2. 

### Evaluation of risk of bias / quality of individual studies

The risk of bias, subjective to the included studies was measured by a customized Quality Assessment Instrument (QAI)^6^ based on QUADAS. This was objectively scored as minimally (scores of 1-12), moderately (13-20) and highly (21-29) sensitive, summarized in Table 1. No minimally sensitive studies were included in the review.

**Table 1:** Inclusion and Exclusion criteria applied for inclusion of studies in the systematic review.

Criteria	Sub criteria	Inclusion	Exclusion
Participants/population	Type of sample	Human studies	Animal studies, in vitro studies
	Age groups	if specified	Not mentioned
	Male to female ratio	if specified	Not mentioned
	Controls	present (either internal /external)	No controls
	Sample size (sample size, not number of teeth studied)	≥5	<5
Intervention(s), exposure(s)	Mediators studied	enzymes [AST, MPO, ALP,βG, LDH, CatB, Cs, cAMP RII, MMPs]	Other than enzymes (cytokines/ hormones/PGs)
	Exposure	Orthodontic force application in healthy patients	Studied in periodontal inflammation/ root resorption/ not related
	Orthodontic mechanics	Specified	Not specified
	Oral hygiene regimen	Mentioned	Not mentioned
	Use of antibiotic/anti-inflammatory drugs	Not used	Not mentioned/ used
	Medium of study	GCF	Other than GCF/ peri-implant fluid/saliva
	GCF sample collection instrument	Periopaper/micropipette/ endodontic paper	Not mentioned

AST: aspartate transaminase, MPO: myeloperoxidase, ACP: acid phosphatase, ALP: alkaline phosphatase, βG: β glucuronidase, LDH: lactate dehydrogenase, CatB: cathepsin B, Cs: caspase, cAMP RII:cyclic adenosine monophosphate (AMP)-dependent protein kinase subunit (RII), PGs: prostaglandins, MMPs: matrix metalloproteinases.

## RESULTS

Were identified 102 articles in Pubmed, 460 in Scopus, 84 in Embase and 6 from hand search, in the initial search. Strict inclusion and exclusion criteria (Table 2) were applied after removing duplicates, resulting in 41 relevant articles. Five studies were further excluded: three studies whose full texts were not retrieved despite contacting the authors repeatedly through mail and academic social networking sites; one was a review on MMPs, and one had sample size smaller than inclusion criteria. Additional exclusion of three studies was done: two with QAI score smaller than 13, and one with a cross-sectional study design (Fig 2).

**Table 2:** Quality Assessment Instrument (QAI) customized from QUADAS (Quality Assessment of Diagnostic Accuracy Studies) tool for assessment of risk of bias for inclusion of studies in the review.

S. No.	Criteria (29)		Response
	Yes	No	Unclear
I. Study design			
1.	Objective: objective clearly formulated		
2.	Sample size: considered adequate		
3.	Spectrum of patients representative of patients receiving the test in practice		
4.	Ethical clearance mentioned		
5.	Selection criteria: clearly described		
6.	Randomization: stated		
7.	Baseline characteristics: clearly defined		
8.	Control: clearly defined		
9.	Orthodontic mechanics explained in sufficient detail to permit replication of experiment		
10.	Orthodontic force: clearly specified		
11.	Description of execution of index test: sufficient to permit replication of test		
12	Absence of time difference between index test & control: mentioned		
13.	Index test executed at specified time and environmental conditions		
14.	Use of proper indices for assessment of gingival & periodontal status (pre-treatment)		
15.	Use of proper indices for assessment of gingival & periodontal status (at each observation time)		
16.	Oral hygiene regime: mentioned		
17.	Prophylaxis done (pre-treatment)		
18.	Prophylaxis done (at each observation time)		
II. Study measurements			
1.	GCF handling characteristics: explained		
2.	Measurement method: appropriate to the objective		
3.	Reliability: adequate level of agreement		
III. Statistical analysis			
1.	Dropouts: dropouts included in data analysis		
2.	Statistical analysis: appropriate for data		
3.	Confounders: confounders included in analysis		
4.	Statistical significance level: P value stated		
5.	Confidence intervals provided		
IV. Study results and conclusions (3)			
1.	Index test compared to baseline		
2.	Index test compared to control		
3.	Conclusions: specific		

*Index test: Refers to collection of GCF at each observation interval in treatment teeth.

PRISMA finally resulted in 48 publications in total, with consensus among all reviewers. The QAI of these studies indicated 13 moderately sensitive and 35 highly sensitive studies.

Data extraction of shortlisted studies^7^ ^-^ ^54^ (for participant characteristics and study design are as follows (Table 3): 

**Table 3:** Participant and study characteristics table.

Reference no.	Sa	M/F	Age	Me	Ix T	cT/gp	Site	Rn	ml	F	t/o f	mc	md/mc	re	to du	No. / ob	tm/ob	B	B=c
7	9	5M/4F	10-18y	IL-1β, βG	1st Mo, 1st PM, CI	NM	MP & MB	N	RME	NM	Im F	RME	Hyrax	Y	81d	10	0, 14, 25, 32, 33, 39, 46, 53, 60, 81 d	14d	Y
8	14	5M/9F	12.5 ± 1.7y	MPO	Single root T	NM	MB & DB	NM	NM	NM	Cn	Lv	Arch wi	N	14d	4	-7d, 0, 2h, 7, 14d	0	Y
9	12	5M/7F	16-20y (17.5± 2.4y)	ALP	Mx C & ct C	1st Mo	Ms C & D 1st Mo	Y	Class I	1st q: 150 cN, 2nd q: 50cN, 100cN, 150cN	Cn	Rt	NiTi sp	NA	3 wk	4	0,1wk, 2wk, 3wk	0	N
10	20	10M/10F	15-25y	ALP	Mx C	ct C	MB, MiB,DB, MP, MiP, DP	Y	Class I bimax	125g	Cn	Rt	NiTi sp	NA	3wk	6	0,1h, 24h, 7d, 14d, 21d	0	N
11	19	5M/14F	16-28y	LDH, AST, TRAP, ALP	Mx C	NM	NM	Y	1st PM Ec	100/150g	Cn	Rt	NiTi sp	NA	5wk	6	0, 1, 2, 3, 4, 5wk	0	Y
12	20	5M/15F	19± 1.3 y	MPO	Md I	NM	MB &DB	N	crw (severe & minm)	NM	Cn	Lv	Arch wi lig	NA	2wk	4	0, 2h, 7d, 14d	0	Y
13	16	6M/10F	13-17y (14 ± 1.67y)	TSP1 MMP9/NGAL	Mx C	ct C	D	NM	1st PM Ec	NM	Cn	Rt	LB	NA	2wk	8	-1h,+1h, 4, 8, 24, 72h, 1wk, 2wk	-1h	N
14	20 (10Clf/10 non Clf)	Clf gp: 7M/3F Non Clf gp: (5M/5F)	15-25y (19.75 ± 2.93y)	ALP ACP AST	Mx I, Mo of same q	NM	NM	N	NM	150cN	Cn	Lv	NiTi wi	NA	30d	5	0, 3, 9, 15, 30d	0	Y
15	20 (10 adol, 10 Ad)	ado - 3M/7F Ad - 4M/6F	ado:14.4 ± 1.43y Ad:28.5 ± 7.83y	MMP-9, RANKL, IL-1 IL-1RA	Mx I	Md I	DB	N	Class I minor crw	NM	Cn	Lv	NiTi wi	Y	20wk	4	0, 3, 6, 18, 20wk	0	Y
16	40 (4gps)	19M/21F	12-18y	LDH	4.1, 4.3 & 4.5	1.1, 1.3 & 1.5.	Bu	NM	Class I Md crw	NM	Cn	Lv	NiTi & thrm wi	Y	42d	6	-2wk, 0, 1h, 7, 28, 42d	-2wk	N
17	16	9M/7F	17.7y	(MMPs) -1, -2, -3, -7, -8, -12, -13	Mx C	ct C	Ms &D	Y	1st PM Ec	150g	Cn	Rt	NiTi sp	N	21d	6	0, 1, 24h, 7, 14, 21d	0	N
18	21	NM	12-20 y	GM-CSF, IFN-?, IL-1β, IL-2, IL-4,IL-5, IL-6, IL-8,IL-10 & TNFα, MMP-9, TIMP-1 & 2, RANKL, OPG	Mx C	2nd Mo	MB &DP	NM	Mx 1st PM Ec	100g	Cn	Rt	NiTi sp	N	42d	4	-10wk, 0, 4h, 7d, 42d	0	N
19	20	10M/ 10 F	15-25y	LDH	Mx C	NM	MB, MiB,DB, MP, MiP, DP	NM	Class I bimax	125g	Cn	Rt	NiTi sp	N	21d	5	0, 1h, 7, 14, 21d	0	Y
20	14	3M/11F	12-28y (18.8 ± 4.8 y)	MMP-3, MMP-9, MMP-13, MIP-1β, MCP-1, RANTES	Mx C	NM	Ms&D	NM	Mx 1st PM Ec	150g	Cn	Rt	V- loop & NiTi sp	N	87d	7	−7d, 0, 1h, 24h, 14, 21, 80d	0	Y
21	99	3 gps; 1st: Non ortho (35M/9F) 2nd;C re (3M/14F) 3rd: Rt (13M/25F)	gp 1:22y10m gp 2: 24 y1m gp 3:20y2m	Cp, cys	gp2: Mx C	gp1	D	NM	gp2: 1st PM Ec	100-150g	Imf	Rt	E Ch	NM	1m	4	0, 1d, 1wk, 1m	0	N
22	11	8F/3M	13-15y (13.9y)	MMP-1, MMP-2	L Mx C	Ag Mx C	MB & DB	N	1st PM Ec	150g	Cn	Rt	NiTi sp	N	8 h	5	0, 1h, 2h, 3h, 4h, 8h	0	N
23	10	5M/5F	M - 22.5 ± 2.8y, F - 23.4 ± 3.9y)	t-PA& PAI -2	M X C	ct & Ag C	D	NM	1st PM Ec	250g	Imf	Rt	Ech	NM	168h	4	0, 1, 24, 168h	0	N
24	10	8F	12-21y	ALP	Mx C	ct C	Ms &D	N	1st PM Ec	100g	Cn	Rt	NiTi sp	N	21d	6	-1, 0, 1, 7, 14, 21d	-1	N
25	9	4M/5F	14.76±2.08y	ALP	Mx 1st PM	NM	MB,DB,P	N	1st PM Ec	50g	Imf	Intr	TMA sp	Y	28d	5	0, 1, 24, 168h, 22d, 28d reac-21d	0	Y
26	17	9F/8M	11-22 y, 16.1 ±3.8 y	LDH	1st Mx Mo	Ag & ct 1st Mx Mo	Ms & D	N	Mo dst	250g	Cn	Rt	NiTi sp	N	21d	4	0, 7, 14 & 21d	0	N
27	5	3F/2M	nov/36	MMP-1 &8	Mx & MdCI/ Mx C	Mx & Md CI	NM	NM		NM	Cn	aln	NM	N	30d	31	0,1-30d,once/d for 1 m	0	N
28	21	11F/10M	11.2-22.5y, 17.17 ±3.3 y	ALP &AST	MxC	Ag & ct C	Ms & D	N	1st PM Ec	150g	Cn	Rt	NiTi sp	N	28d	2	0, 28d	0	N
29	10	5M/5F	22.5±3.9y	Cp B	C	ct & Ag C	D	NM	1st PM Ec	250g	Imf	Rt	E ch	NM	168h	4	0, 1h, 24h, 168h	0	N
Ref No.	Sa	M/F	Age	Me	Ix T	cT/gp	Site	Rn	ml	F	t/o f	mc	md/mc	re	to du	No. / ob	tm/ob	B	B=c
30	16	10F/6M	11-21y, 15.5±3.5y	ALP	Mx 1st Mo	ct &Ag 1st Mo	Ms & D	NM	Mo dst	250g	Cn	dst	NiTi sp	NM	4wk	6	0, 1h, 1, 2, 3, 4wk	0	N
31	9	5M/4F	10-18y	IL-1β, βG	Mx 1st Mo, 1st PM & CI	NM	MB &MP	NM	Mx cst	NM	ImF	RME	Hyrax	Y	74d	10	BL-0 (B,O1), 14d (c,O2) Al-4d (O3), 11d (O4), 12d (O5), 18d (O6), 19d (O7), 25d (O8), 32d (O9), 39d (10), 60d (O11)	0	N
32	12	3M/9F	14.4±0.9 y	IL-1β, IL-6, TNF-α, EGF, β2-µG	C	Ag C/ct C	D	NM	1st PM Ec	250g	Imf	Rt	E ch	N	7d	4	0, 1, 24, 168h	0	N
33	9	NM	13-17 y	TRAP5b, IL-10,TNF-α	Mx & MdC	ct C	MB, MiB, MP & DB, MiP, DP	NM	1st PM Ec	150g	Cn	Rt	NiTi sp	NM	28d	5	0, 1h, 24h, 7d, 28d	0	N
34	19	13F/6M	16 - 28y	ALP, AST, TRAP	R & L Mx C	NM	D	NM	Mx 1st PM Ec	gp1-100g gp 2-150g	Cn	Rt	NiTi sp	NM	5wk	6	0, 1wk, 2wk, 3wk, 4wk, 5wk	0	Y
35	12	NM	14-24 y	LDH	Mx C	NM	Ms & D	NM	Class II C	1N & 1.5 N	Cn	Rt	NiTi sp	NM	5wk	6	0wk, 1wk, 2wk, 3wk, 4wk, 5wk	0	Y
36	12	11F/1M	14-24y	TRAP	Mx C	BS	Ms &D	Y	1st PM Ec	100/150g	Cn	Rt	NiTi pushsp	N	5wk	6	0, 1, 2, 3, 4, 5wk	0	Y
37	14	4M/10F	15-27y	ALP	Mx C	NM	Ms &D	NM	crw (4-8mm)	NM	Cn	lv, aln	NiTi wi	N	3wk	4	0, 1, 2, 3wk	0	Y
38	10	8F/2M	15-27y	ALP	Mx C	NM	Ms &D	NM	1st PM Ec	150g	Cn	Rt	NiTi pushsp	N	12wk	5	0, 1, 4, 8, 12wk	0	Y
39	13	NM	14.4±3.7y, 23.3±4y	AST	Mx C	ct C	Ms &D	NM	1st PM Ec	100g	Cn	Rt	NiTi pushsp	N	12wk	5	0, 1, 4, 8, 12wk	0	N
40	13 (6ado, 7Ad)	NM	14.4±3.7y, 23.3±4y	AST	Mx PM	Ag PM	Ms &D	NM	1st PM Ec	50-75g	Cn	Lv	NiTi wi	Y	28d	5	0, 7, 14, 21, 28d	0	N
41	22	12F/10M	13-22y	AST	Mx C	NM	Ms & D	Y	NM	NM	Cn	Lv	NiTi wi	NM	6m	5	0, 1wk, 1m, 3, 6m	0	Y
42	12	7F/5M	14±2y	ACP, ALP	Mx C	Ag C, ct C	Ms & D	Y	1st PM Ec	250g	Cn	Rt	NiTi open coil sp	NM	28d	3	0, 14, 28d	0	N
43	10	5F/5M	15-20y	ALP	MxC, Mx 2nd PM	NM	D of C & Ms of 2nd PM	NM	1st PM Ec	150g	ImF	Rt	E ch	NM	28d	6	0, 1, 7, 14, 21, 28d	0	Y
44	23	15F/8M	9±1.4y	ALP	Mx rt & lt 1st M	Ag ist M	MB, MiB, DB, MP, MiP, DP	NM	Mx constr	16N/turn	ImF	Mx Exp	Hyr	2/d	6m	3	0, 3, 6m	o	N
45	10	7F/3M	14 - 27 y	ALP	Rt Mx C	Lt Mx C	Ms, D	NM	1st PM Ec	250g	Cn	Rt	NiTi sp	NM	4wk	6	0, 1h, 7, 14, 21, 28d	0	N
46	7	5F/ 2M	14 - 27 y	ACP	Rt Mx C	Lt Mx C	Ms, D	NM	1st PM Ec	NM	Cn	Rt	NiTi sp	NM	4wk	6	0, 1h, 7, 14, 21, 28d	0	N
47	20	9F/11M	12- 25 y	LDH	MxC	ct C	MB, MiB, DB, MP, MiP, DP	NM	1st PM Ec	125g	Cn	Rt	NiTi sp	NM	21d	6	0, 1h, 1, 7, 14, 21d	0	N
48	18	10F/8M	nov/22	AST	Max 1st Mo	ct & Ag 1st Mo	Ms & D	N	Mo dst	250g	Cn	Rt	NiTi sp	N	4wk	6	0, 1h, 1, 2, 3, 4wk	0	N
49	20	6F/4M	20.6 ± 3.2y	ALP	Mx C, Md C	BS	D	Y	1st PM Ec	200 cN	Imf vs Cn	Rt	Hycon, TieB	Screw 2/wk	28d	6	0, 1hr, 7, 14, 21, 28d	0	Y
50	55	28F/ 27M	15.1 (1.7)	Adiponectin, Leptin, Resistin, MPO, CRP, MMP 8,9, TIMP1, MMP8/TIMP1, MMP9/TIMP1,RANKL	Mand 6 anterior teeth	Normal weight children	D	NM	Non Ec	NM	Cn	Aln	NiTi wi	NM	Completion of Aln	4	0, 1h, 1wk, completion of Aln	0	N
51	22	14F/8M	11-21y	ALP	Max 1st - M rt &Lt	Mand 1st - M rt & Lt	NM	MB	Exp	400g	Imf	Mx Exp	Hyr	1/3m	1y	4	0, 2wk, 4wk, 1y	0	N
52	60	41F/19M	18 ± 1.5	MPO	Mand CI	BS	NM	NM	4-6mm mand I crow	NM	Cn	Aln	MSSS, HANT, SE wi	N	14d	4	0, 2h, 7, 14d	0	Y
53	45	NM	6.25, 5.6, 6.10	MPO	Mand I	BS	NM	Y	4-6mm mand I crow	NM	Cn	Aln	MSNiTi, HANT, SE wi	N	14d	4	0, 2h, 7, 14d	0	Y
54	30	NM	9-15y	AST	Rt Mx PM	Lt Mx PM	NM	NM	NM	NM	Cn	Aln	NiTi wi	N	4wk	6	0, 1h, 1, 2, 3, 4wk	0	N

A-article, f-force, t/o-type of, mc-mechanics, md/mc-mode of mechanics, tm- time, a-appliance, re-reactivation, to-total, du-duration, n-number, ob-observation, B-baseline, min- minutes, g- grams, Ir- Interrupted, Cn- Continuous, Im- intermittent, Rt-retraction, sg-segmented, sp-spring, Ech-elastomeric chain, NiTi-nitinol, c-control, NM-not mentioned, y-year, d-day, m-month, h-hour, lv-levelling, se-separator, ac-activated, HG-headgear, NHG-non-headgear, bu-buccal, la-labial, RME-rapid maxillary expansion, HR-hybrid retractor, RCD- rapid canine distaliser, Sa-Sample, M/F-male/female, E- enzyme, Me- mediator, T-tooth, sc-specification, rn-randomisation, ml-malocclussion, HS-Handsearched, P-Pubmed, S-Scopus, GS- Google scholar, N-No, Y-yes, Mx-Maxilla, Md-Mandible, H-history, ls-loss, gv-gingival, if-inflammation, PD-probing depth, wk-week, R-right, L-left, C-canine, PM-premolar, Mo-molar, CI-central incisor, I-incisor, Ag- Antagonistic, ct- Contralateral, ip-interproximal, op-opposing, Ex- Experimental, c- Control, aj-adjacent, Exs-Experimental site1, Ec- Extraction, Ms- Mesial, D- Distal, rq-required, q-quadrant, OTM-orthodontic tooth movement, sf- surface, ado-adolescent, AST-aspartate transaminase, TRAP-Acid phosphatase, ALP-Alkaline Phosphatase, βG- beta glucuronidase, MMP-matrix metalloproteinase, LDH-lactate dehydrogenase, Cp-Cathepsin, MPO- myeloperoxidase, CK-creatinine, NO-Nitric oxide, IL-Interleukin, CRP- C Reactive Protein, hm-humidity, sc-specification, ins-insertion, MB-Mesio-buccal, ML-Mesio-lingual, DP-Disto-palatal, DB- Disto-buccal, df-differentiation, gp-group, cmp-compression, kPa-kilopascal, mx-maximum, gw-growth, Oc-osteoclast, Ix- Index, Bu Tp-buccal tipping, C-canine, Clf-cleft, I-incisor, NA-not applicable, wk-week, crw-crowding, minm-minimum, bimax-bimaxillary, wi-wire, lig-ligature, Ad-adult, RANKL-receptor antagonist nuclear kappa ligand, OPG-osteoprotegerin, IL-_1RA-interleukin 1 receptor antagonist, therm-thermoplastic, t-PA-plasminogen, TNFα-tumour necrosis factor, TIMP-Tissue inhibitor metalloproteinase, MCP- Methyl-accepting chemotaxis protein, MPO-myeloperoxidase, ortho-orthodontic, cys-cysteine, cN-centinewton, TSP-thrombospondin 1, NGAL-neutrophil gelatinase-associated lipocalin, ACP-acyl carrier protein, CS-chondroitin sulphate, GM-CSF- Granulocyte-macrophage colony-stimulating factor, IFNγ-Interferon gamma, MIP-Macrophage inflammatory protein, βG-beta globulin, PAI-plasminogen activator inhibitor, EGF-Epidermal growth factor, dst-distalisation, Intr-intrusion, aln-alignment, cst-constriction, AL-after loading, BL-before loading, Mx constr- Maxillary constriction, Exp- Expansion, Hyr- Hyrax, LB-laceback, TB-Tie back, SE-superelastic NiTi, HANT- heat-activated NiTi, MSSS- multistranded stainless steel.

» Sample size: Sample size was categorized in three groups, ≤15 (n=22), 15-20 (n=15), ≥21 (n=10) and one study each having sample of five subjects^27^ and 99 subjects.^21^
» Sex predilection: Forty- one studies mentioned sex distribution in the sample, two of which had female subjects only,^24^ ^,^ ^36^ and five had equal numbers of male and female subjects.^10^ ^,^ ^19^ ^,^ ^23^ ^,^ ^29^ ^,^ ^43^
» Age predilection: Studies used age as either range or mean with standard deviation in all studies; one study considered two separate age groups of adolescents and adults.^15^
» Number of studies reporting enzymes: Alkaline phosphatase was evaluated in maximum number of studies (n=17), closely followed by AST in 10, matrix metalloproteinases (MMPs) in eight, LDH in six, MPO in five and TRAP in four and acid phosphatase (ACP) in three studies. Two studies studied βG, cathepsin (Cp) and tissue inhibitor of MMPs (TIMPs) each. Single studies evaluated cystatin (Cys) and thrombospondin1 (TSP1). Additionally, granulocyte-macrophage colony-stimulating factor (GMCSF), epidermal growth factor (EGF), macrophage inflammatory protein-1β (MIP-1 β), methyl-accepting chemotaxis protein-1 (MCP-1), chemokine RANTES (Regulated on activation normal T cells expressed and secreted) were evaluated as secondary outcomes.
» Study duration: The duration of studies ranged from 8 hr to 24 weeks (wk) to the maximum of one year (y). One study each was done for 8hr, 1wk, 5month (m) and 1y duration, two studies for 6m, three for 2m, five each for 2wk and 3m, eight for 3wk, 15 for approximately 1m. One study did not specify duration - only completion of alignment.
» Observation intervals for GCF collection: Studies had GCF collection at repeated observation time points (OTP) ranging from 2 times^28^ to 31 times (each day of the month).^27^ Six OTPs were taken in 16 studies, closely followed by 4 OTPs in 15 studies, 9 OTPs in nine studies, 3 and 10 OTPs in two studies each, 2, 7, 8 and 31 OTPs in single study each.
» Site for GCF collection: Forty one studies specified mesial or distal or buccal site for GCF collection while seven studies mentioned the tooth but not the site for sample retrieval. The technique by Lamster et al.^55^ utilizing six sites was used in four studies.^10^ ^,^ ^19^ ^,^ ^33^ ^,^ ^44^ ^,^ ^47^
» Mechanics of force: Studies used continuous force both for tooth retraction (26 studies) and leveling of arches (13 studies). Retraction involved 19 studies using NiTi coil spring, two using steel ligature lacebacks, three using NiTi push coil spring, and one study each for V loop and NiTi open coil spring. Besides, nine studies used intermittent orthodontic/orthopaedic forces, employing elastomeric chain for retraction in five, Hyrax for expansion in three, and TMA spring for intrusion in one study.
» The level of force: Only 33 studies mentioned force levels for OTM. The level of forces ranged from 50g, 50-75g, 100-150g, 16N/turn, 1-1.5N, 200cN, 400g in one study each, 125g in three, 100g in six, 250g in eight and 150g in seven studies. Few studies had different treatment groups employing variable magnitudes of force.^9^ ^,^ ^11^ ^,^ ^34^ ^,^ ^35^ ^,^ ^36^

### Oral hygiene regimen and gingival health assessment (Table 4)

**Table 4:** Oral hygiene regimen.

Ref No.	Oral px (Pre t/t)	Oral px (Every ob po)	Oral hy instr/motiv	Mw	fq/o mw/d	asm for gv & pd in (pre t/t )	At every ob po
7	Y	NM	Y	Cx glu	2	Y	Y
8	Y	NM	NM	NM	NM	Y	NM
9	NM	NM	Y	0.15% Benz HCL/d	1 /d	NM	NM
10	Y	NM	Y	0.5 oz of 0.2% cx glu	2/d	NM	NM
11	Y	NM	NM	NM	NM	Y	NM
12	NM	NM	NM	NM	NM	NM	NM
13	Y	Y	Y	NM	NM	NM	NM
14	Y	NM	Y	NM	NM	Y	Y
15	Y	NM	NM	NM	NM	Y	NM
16	Y	Y	Y	NM	NM	Y	Y
17	Y	Y	Y	0.5 oz of 0.2% cx glu	2/d	Y	Y
18	NM	NM	NM	NM	NM	Y	Y
19	Y	Y	Y	0.5 oz of 0.2% cx glu	Y	Y	Y
20	Y	Y	Y	0.12% cx glu	2 /d for 4 wk	NM	NM
21	NM	NM	Y	NM	NM	Y	NM
22	NM	NM	Y	NM	NM	NM	NM
23	Y	Y	NM	NM	NM	Y	Y
24	Y	Y	Y	cx glu	2/d	Y	NM
25	NM	NM	NM	Benz HCL	NM	NM	NM
26	Y	NM	Y	NM	NM	Y	Y
27	NM	NM	NM	NM	NM	Y	NM
28	Y	NM	Y	NM	NM	Y	Y
29	NM	NM	NM	NM	NM	Y	NM
30	NM	NM	Y	NM	NM	Y	Y
31	Y	NM	Y	cx glu	NM	Y	Y
32	NM	NM	NM	NM	NM	Y	Y
33	Y	NM	Y	NM	NM	Y	Y
34	NM	NM	Y	NM	NM	NM	NM
35	Y	NM	Y	NM	NM	NM	NM
36	Y	NM	NM	NM	NM	NM	NM
37	Y	Y	Y	N	N	Y	Y
38	Y	Y	Y	N	N	Y	Y
39	Y	Y	Y	N	N	Y	Y
40	Y	Y	Y	N	N	Y	T
41	Y	Y	Y	N	N	Y	Y
42	NM	NM	Y	cx glu	2 /d	Y	Y
43	Y	NM	Y	N(against it)	N	Y	NM
44	Y	Y	Y	0.012% cx glu	2/d	Y	Y
45	Y	Y	Y	Cx glu	NM	Y	Y
46	Y	Y	Y	NM	NM	Y	Y
47	Y	NM	Y	NM	NM	Y	NM
48	Y	NM	Y	NM	NM	Y	Y
49	NM	NM	NM	NM	NM	NM	NM
50	NM	NM	NM	NM	NM	Y	Y
51	Y	Y	NM	NM	NM	Y	NM
52	Y	NM	Y	NM	NM	Y	NM
53	Y	NM	Y	NM	NM	Y	NM
54	Y	NM	NM	NM	NM	Y	NM

A-article, Mw-mouth wash, fq/o-frequency of, d-day, px-prophyaxis, t/t-treatment, ob-observation, po-point, asm-assessment, gv-gingival, pd-periodontal, in-inflammation, cx glu-chlorhexidine gluconate, Y-yes, NM-not mentioned, N-no, h-hour, Benz HCL-benzydamine hydrochloride, wk-week, hy-hygiene, instr-instructions,motiv-motivation.

### GCF characteristics (Table 5)

**Table 5:** GCF characteristics.

Ref No.	Time	tp	hm	mt/o cl	ins (in mm)	du/o mm	rep mm	i/o mm	mt/o mm	tp of st	mt/o al	pr cc
7	NM	30%	21°C	PP	NM	30s	NM	NM	PT6000	NM	ELISA	pg /30-s
8	NM	NM	NM	PP	NM	30s	4	NM	NM	-70°C	SP	PMNs/µl
9	NM	NM	NM	PP	1mm	1min	3	1min	NM	NM	SP	IU/1 µl
10	NM	NM	NM	PP	NM	1min	NM	NM	PT 8000	-70°C	SP	IU/L
11	NM	NM	NM	PP	1-2mm	1min	3	NM	NM	-20°C	ELISA	LDH, AST-mIU/ml, TRAP, ALP-ng/ml
12	NM	NM	NM	PP	NM	30s	4	NM	NM	-70°C	SP	U/100 ml
13	NM	NM	NM	PP	NM	30s	3	1min	PT 8000	-20°C	ELISA	ng/ml
14	NM	NM	NM	µP	NM	5min	NM	NM	NM	-70°C	SP	U/µl
15	NM	NM	NM	PP	NM	1min	NM	NM	NM	-80°C	QAK	pg/ml
16	NM	NM	NM	PP	1mm	30s	NM	NM	NM	-30°C	SP	µg / ml
17	NM	NM	NM	PP	NM	30s	NM	NM	NM	-70°C	IA	pg/site
18	NM	NM	NM	PP	NM	30s	NM	NM	PT 8000	NM	LMAT	pg/ml
19	NM	NM	NM	PP	NM	1min	5	NM	NM	NM	SP	µmolU/L
20	NM	NM	NM	PP	1mm	NM	NM	NM	PT 8000	−80°C	mb-IA	pg/site
21	NM	NM	NM	PP	NM	30s	NM	NM	PT8000	-80°C	Flr	Cp; µU/ µl, Cys; ng/µl
22	9am	20°C	40%	PP	NM	30s	NM	NM	NM	-70°C	WB	NM
23	NM	NM	NM	PP	1mm	1min	2	1min	PT8000	-30°C	ELISA	µg/µl
24	NM	NM	NM	µP	NM	NM	NM	NM	NM	-70°C	NM	NM
25	NM	NM	NM	PP	NM	1min	2	5s	NM	-80°C	ELISA	pmol/mg
26	NM	NM	NM	PP	1mm	30s	NM	NM	NM	-80°C	SP	mU
27	NM	NM	NM	PP	NM	3min	NM	NM	NM	-20°C	WB	µg/l
28	NM	NM	NM	PP	1mm	10s	NM	NM	NM	NM	SP	mU/sample
29	NM	NM	NM	PP	1mm	1min	1	30s	NM	-30°C	WB	pU/µl
30	NM	NM	NM	PP	1mm	NM	NM	30s	NM	NM	SP	mU/sample
31	NM	21°C	30%	PP	NM	NM	NM	30s	PT6000	-70°C	ELISA	U/30-s GCF sample
32	NM	NM	NM	PP	1mm	1min	1	30s	PT	-30°C	ELISA	pg/µg
33	NM	NM	NM	PP	NM	NM	NM	30s	PT8000	-20°C	ELISA	pg/µL
34	NM	NM	NM	PP	1-2mm	1min	2	1min	NM	NM	SP	µmol/ min
35	NM	NM	NM	PP	1mm	NM	NM	1min	NM	NM	SP	U/mg
36	NM	NM	NM	PP	1mm	1min	3	1min	NM	NM	SP	U/mg
37	NM	NM	NM	end PP	1mm	30s	3	90s	NM	-40°C	SP	µmol/min
38	NM	NM	NM	end PP	1mm	30s	3	90s	NM	-40°C	SP	µmol/min
39	NM	NM	NM	end PP	1mm	30s	3	90s	NM	-40°C	SP	µmol/min
40	NM	NM	NM	end PP	1mm	30s	3	1min	NM	4°C	SP	µmol/min
41	NM	NM	NM	µP	2mm	NM	NM	NM	NM	-70°C	SP	U/mg
42	NM	NM	NM	PP	1mm	30s	NM	NM	NM	-20°C	SP	NM
43	Y	NM	NM	FP	NM	1min	NM	5s	NM	-80°C	PNPP kin	NM
44	NM	NM	NM	FP	1mm	30s	NM	NM	NM	-80°C	SP	mU/sample
45	NM	NM	NM	µP	NM	NM	NM	NM	NM	NM	SP	U/L
46	NM	NM	NM	µP	NM	NM	NM	NM	NM	NM	SP	U/L
47	NM	NM	NM	µP	NM	NM	NM	NM	NM	-80°C	SP	µmol units/L)
48	NM	NM	NM	pp	1mm	30s	NM	NM	NM	NM	SP	mU/sample
49	NM	NM	NM	µP	NM	NM	NM	30s	NM	NM	SP	IU/L
50	Y	NM	NM	PP	1mm	30s	NM	NM	PT8000	-80°C	SP	pg/mL
51	NM	NM	NM	end PP	1mm	30s	NM	NM	NM	-30°C	SP	mU/sample
52	NM	NM	NM	PP	NM	30s	4	30s	NM	-70°C	SP	units/100 µL
53	NM	NM	NM	PP	NM	30s	2	30s	NM	-70°C	SP	units/100 µL.
54	NM	NM	NM	pp	1mm	1min	NM	NM	NM	NM	SP	mU/s

A-article, tp-temperature, hm-humidity, mt/o-method of, cl-collection, sp-specification,i ns-insertion, mm-millimeter, du/o-duration of, rep-repeated, i/o-interval of, st-storage, al-analysis, pr-protein, cc-concentration, NM-Not Mentioned, N-No, Y- Yes, PP- Periopaper, PT- Periotron, WB- Western Blot, ELISA- Enzyme linked immunos orbent assay, IA- Immunoassay, RIA- Radio IA, meas-measurement, pg-picogram, µg-microgram, ml-millilitre, µL-microlitre, GCF-gingival crevicular fluid, tot-total, g-gram, ng-nanogram, s-second, min- minutes, ^0^C-degree Celsius, SP-spectrophotometery, Ar-array, As-assay, mb-multiplex bead, LMAT-Luminex multianalyte techonology, QAK- Quantibody Ar kit, end PP-endodontic paperstrip, FP- Filter paper strips, µP-micropippete, IU-international units, L-litre, LDH-lactate dehydrogenase, AST-aspartate transaminase, TRAP-Acid phosphatase, ALP- Alkaline Phospahatase, PMNs -polymorphonucleosides, Cp -cathepsin, Cys -cysteine, Tot -total, pmol -picomol, flr-flurometery, QA- Quantibody assay, PNPP kin- para nitrophenyl phosphate kinetic, pp- paper point.

» GCF collection: GCF was collected by Periopaper (OraFlow, Plainview, New York, NY, USA) in 32 studies, micropipette in seven, filter paper in two, paper point in two and endodontic paper strip in five studies. Time of sample collection, room temperature and humidity conditions were specified in three studies each.

» GCF handling: Depth of Periopaper insertion was 1mm in 21 studies, 1-2mm in two, and 2mm in one study. Duration of GCF collection was 30 seconds (s) in 21 studies, 60s in 13 studies and 10s, 3 minutes (min) and 5 min in one study each. GCF measurements were repeatedly taken in 18 studies with specified number of intervals, interval of repeat measurements were 30s (n=8), 60s (n=7), 90s (n=3) and 5s (n=2). Storage of samples was done at -20^o^C (n=5), -30^o^C (N=4), -40^o^C (n=3), -70^o^C (n=11) and -80^o^C (n=9). Retrieval of GCF from Periopaper was done by Periotron (OraFlow, PlainView, New York, NY, USA) in 11 studies, but not mentioned in 38 studies. Enzymes levels were estimated by ELISA (n=8), spectrophotometry (n=30), immunoassay (n=2), Luminexmultianalyte technology (n=1), Quantibody Array kit (n=1), western blotting (n=3), fluorometry (n=1) and para-nitrophenol phosphate kinetic (n=1), but omitted in one study. Protein concentration in GCF was measured in variable units in 38 out of 42 studies.

## DISCUSSION

The findings of the current review are presented in Table 6. It depicts various enzymes released in GCF in a time-dependent manner and also establishes correlations (if any) with levels or type of force applied. In this review, we have tried to establish associations of enzyme levels to magnitude or type of force in each phase of OTM, given by Burstone^56^ in his classic model or four phase time/displacement modification model.^57^ ^,^ ^58^

**Table 6:** Differential expression of enzymes in GCF.

Ref No.	sts al ap	cf	Drop outs	Up / down rg	Pk	sd oc	cr	sts sn rd
7	1-tailed paired Student t	Y	NM	βG: inc	M-010 PM-07 CI-08	IL-1β sign inc for Mo- O5 to O10 for PM-O6 to 010 For CI-04, 06, 07, 09, 010 & dec at O2 for Mo, PM, CI	stronger F cause higher levels of IL-1β & βG	βG inc for Mo- 07 to 010 PM-07, 08, 010 CI-06, 07, 010 & dec at O2 for Mo, PM, CI
8	ANOVA, paired t test	Y	NaM	Inc at 2h, bas in 7d	2h	Inc MPO in saliva at 2h, B in 7d	+ve cr of lvl in GCF & saliva	Inc at 2h
9	(ANOVA) Kolmogorov-Smirnov test, Paired-samples test	Y	N	Inc	2wk	NM	NM	In cns F: Lvl pk at 2wk In gradually inc F: Lvl pk at 3wk
10	Kolmogorov and Smirnov (ANOVA) & Tukey’s post-hoc test	Y	N	Inc	14d	GCF vol inc from 0 - 21d Sn inc at 14d	Exp si; lvl inc on 14d cr with pk in GCF vol	Exp si; pk at 14d
11	Paired t test Pearson’s cr	Y	N	LDH inc at 2, 3& 4 wk(100 g) & 1, 2 & 3wk (150 g). AST inc at 4 & 5wk (100 g) & 3 & 4wk (150 g). TRAP inc at 5wk (100 g)	AST: 1wk TRAP: 2wk ALP: 5wk	In saliva:AST inc at 5wk, TRAP at 2wk, ALP at 1 to 5wk	Weak cr b/w enz quantity & activity	LDH inc at 2, 3 & 4 wk (100 g) & 1, 2 & 3wk (150 g). AST inc at 4 & 5wk (100 g) & 3 & 4wk (150 g) TRAP inc at 5wk (100 g)
12	Friedman test for intergp & intragp, Wilcoxon test for related samples, Kruskal-Wallis test for independent samples in both gps	Y	N	inc	2h	minm & severe crw: inc from 0 at 2h, 7d, 14d in saliva	No cr of crw with change in MPO	At 2h, 7d sn inc from B & 14d
13	Intra gp: Friedman’s test, Wilcoxon test Inter gp: Mann-Whitney U test, Pearson’s test	Y	N	inc	MMP9: 8h MMP9/NGAL: 72h	TSP1: inc from B at 8h to 72h, dec at 1wk	Strong & sn cr b/w MMP9/NGAL & TSP1 in IxT	MMP9: inc from B at 4h, 8h, 1wk, 2wk. MMP9/NGAL: inc from B at 8h,24h, 72h
14	Intergp: Mann Whitney U test. Intra gp: Students unpaired t-test	Y	N	Inc	ACP: 3d ALP, AST: 15d	NM	NM	Inter gp; ACP: pk at 3d ALP, AST: pk at 15d Intra gp: ACP, ALP, AST higher in Mx I than Mo
15	SAS version 9.2 proc mixed subroutine	Y	N	No sts sn change	NM	Exp si: in Ad, IL-1/1L-1RA dec in 3wk aftr 1st wi lig ado, RANKL/OPG pk at 6wk aftr 1st rect wi lig	NM	b/w Exp & cT; no sts sn change B/w Ad & adol: no sts sn diff
16	MedCalc software Intergp: Student’s t-test, ANOVA.	Y	N	SL+ NiTi wi: inc SL+ thrm wi: dec	No sts sn change	Visual pl scr dec sts sn	NM	No sts sn change b/w Exp & cT or within each gp
17	Luminex analysis	Y	N	MMP1,3:inc, pk at 24h MMP8: pk at 14d	24h	NM	NM	No sts sg diff b/w comp & tn, MMP1, 3: inc, pk at 24h MMP8: pk at 14d
18	Paired non-parametric Kruskall-Wallis. Spearman Rank Sum anal	Y	NM	Inc	Exp-TIMP, MMP-9:4h cmp: TIMP-1, MMP-9:4h, TIMP-2: 7d	+ve cr of GCF vol & PI at 0 at tn, cmp +ve cr of TNF-α, IL-1β, IL-8, GM-CSF to speed of OTM at 4h in Exp cr of IL-1β, IL-8, TNF-α inc to if at 0 at cmp	+ve cr of MMP-9 & TIMP to speed of OTM at 4h in Exp	TIMP1 & 2, MMP-9 inc at 4h in Exp
19	GraphPad®Instat, ANOVA, Friedman	Y	NM	Inc	14d, 21d	NM	NM	Inc at 7, 14, 21d
20	Friedman, Mann-Whitney	Y	NM	Inc	1h	GCF vol higher in cmp than tn at 21d	NM	MMPs inc at 1h, dec at 24h
21	ANOVA		NM	Cp: dec Cys:inc	1d	GCF vol inc in 1d, dec at 1m	-ve cr in Cp & Cys Lvl	Cp;dec at 1d, inc to B in 1m Cys;inc in 1d, dec to B in 1
22	ANOVA	Y	NM	Inc	MMP1-1h MMP2-1h,8h	NM	NM	Inc in MMP1 (tn) -1h-3h, (cmp) -1h Inc in MMP2 (tn) - 1h, (comp)-8h
23	Mann-Whitney U-tests	N	NM	Inc at 24h at Ix t >cT	24h	GCF vol no sn diff at 24h	NM	Inc at 24h
24	ANOVA &LSD	Y	NM	Inc	14d	NM	NM	Inc in ALP -7, 14 at Ms & D
25	Friedman test	Y	NM	Dec	I d	Dpd, osteocalcin dec	NM	Dec from 0 to 28 d, inc on 7d
26	Friedman and Bonferroni-corrected, Wilcoxon paired signed rank tests	Y	NM	Inc	14d	NM	NM	ct gp greater than Ag gp on 14 d & 21d
27	NM	Y	NM	Lvl of MMP-8 inc in Ix t>c	NM	NM	NM	Lvl of MMP-8 inc 12 times in Ix t>c
28	Friedman & Bonferroni-corrected, Wilcoxon paired signed rank tests	Y	NM	Inc	28d	Aa colonization inc sn on 28d in ExpT & ct gp	NM	Inc at 28d in Ix T as compd to Ct T & Ag T
29	Friedman & Bonferroni-corrected, Wilcoxon paired signed rank tests	Y	NM	Inc	tn:7d cmp:7&14d	NM	NM	AST: inc in Ix T & ct T as compd to Ag T, inc in IxT as compd to ctT on tn si on 14d & on cmp on 7d & 14d, inc in IxT on cmp than tn on 7d
30	Mann Whitney U-test	Y	N	Inc	24h		NM	CpB higher at 24h at IxT
31	Bonferroni-corrected, 1-way repeated measures ANOVA, paired Student t test	Y	NM	Inc	14d	NM	NM	Sign inc on both M & D at 1,2,3,4 wks
32	One-tailed paired Student t test	Y	N	Inc	βG -25d IL-1β-	sn inc in IL-1β level at 4d &19d to 60d AL	NM	inc in βG at 25d to 60d AL
33	Student’s t test	Y	N	IL-1β, IL-6, TNF-α, EGF, β2-µG	inc	24h	Intra-grp in Exp: IL-1β inc at 24h > BS,IL-6 inc at 24h > BS or 168h,TNF-α inc at 24h > BS or 168h, EGF inc at 24h>BS Intergrp btw cont & Exp: IL-1β inc in Exp>cont at 24h, Mean IL-6 in Exp >ant cont, TNF-αin Exp at 24h> ant cont, EGF in Exp at 24hr> ant cont	NM
Ref No.	sts al ap	cf	Drop outs	Up / down rg	Pk	sd oc	cr	sts sn rd
34	ANOVA	Y	NM	Inc	7d	TNF-α in D & Ms sites of TT sn higher than both sites of c, also >.B, inc sn at 1 h & 24h. IL-10 dec during Exp period at c & TT	NM	TRAP5b Level in D & Ms sites of TT were sn higher than that at both sites of cT compd with B values, inc was sn at 1 h & 24h.
35	Shapiro-Wilk test	Y	NM	inc	100g gp-TRAP-3wk 150g gp-ALP & TRAP -5wk	NM	NM	In 100 g gp, TRAP sn inc in 3-5 wk compd to TRAPB. ALP & AST slightly inc. In 150 g gp, ALP & TRAP slightly inc compd with their B. AST sn inc in 5 wk.
36	Student’s paired t test	Y	NM	Inc	M:4wk D:1.5N-2wk	NM	NM	LDH at Ms site in 1.0 N &1.5 N gp, inc sn on 4th wk. At D site, LDH with 1.5 N was higher than 1.0 N throughout 5 wk of TM. LDH with 1.5 NF inc at both Ms (wk 2) & D site (wk 3) with sn diff to 1.0 N F
37	Kruskal Walis test.	Y	NM	TRAP inc in 150g F: Ms site peak 3wk, D site Pk 4wk den dec 100g F: Ms site 2wk Pk den dec, D site 5wk 4wk: Lvl in 150g> 100g F (D site) 5wk: Lvl in 100g>150g F (Dsite)	150g: D site -4wk, Ms site-3wk 100g:D site-5wk. Ms site-2wk	Rate of OTM at 150g>100g	150g F at 3 & 4wk>100g f, +ve cr of Lvl of TRAP & rate of OTM	150g gp, Ms si: inc at 3wk>BS At D si: inc at 4k>BS TRAP at 150 gm>100gm F at 4wk (D site)
38	Paired sample t-test	Y	NM	inc, pk at 2wk, at D >Ms- 1wk	Ms, D Si: 2wk	NM	NM	Inc at 1wk, 2wk from Bas
39	Paired sample t-test	Y	NM	Dec: 1wk,4wk on Ms, D si	4wk	NM	-ve cr of amt of OTM & Lvl of ALP	Dec at 4wk
40	Wilcoxon signed rank test	Y	NM	Inc: at 1wk,4wk, stabilised	4wk	NM	NM	Inc at 1wk, 4wk, At D si>Ms si
41	Wilcoxon signed rank test	Y	NM	Inc at 1wk, dec in next 3wk	1wk	N	NM	Pk at 1wk at Ix T>cT
42	unpaired and paired t-test	Y	NM	Inc at archwi >self lig site, inc at 1wk	1wk	Bac count in archwi>self lig	+ve cr in self lig & AST Lvl	Inc at archwi >self lig
43	ANOVA, paired t-test using SPSS	Y	NM	ALP, ACP inc at 14d, 28d, ALP at Ms>D, ACP inc in Ms &D	ALP, ACP : 14d	NM	NM	ALP, ACP inc, ALP inc more on M si
44	ANOVA, Student's t-test	Y	NM	ALP dec, D of C > Ms of 2nd PM on 1, 7, 14, 21, 28d	Dec	NM	NM	Dec at D of C > Ms of 2nd PM on 1, 7, 14, 21, 28d
45	Friedman test followed by a Bonferroni-corrected Wilcoxon paired signed rank test	Y	Y	ALP inc in 3m, 6m	Inc	PD with ALP actv	+ve corr of ALP lvl with time at tn si	ALP at 3m, 6m > cT
46	ANOVA,Tukey’s HSD Post-Hoc test, Mann-Whitney U-test	Y	NM	ALP inc 14d, 28d	28d	NM	NM	ALP at Ms si of TT>CT at 14d, 28d At Ms si>Dsi at 14d, 28d
47	ANOVA, Independent Samples t-test, Mann- Whitney U-test	Y	NM	ACP inc both Ms & D si D si>M si at 7d, 21d	21d	NM	NM	D si>M si at 7d, 21d TT>cT at 7d, 21 d at Dsi
48	ANOVA,Tukey HSD	Y	NM	LDH inc at TT>cT 7d, 14d,21d	28d	NM	NM	LDH inc frm 7d-14d at TT, TT>cT at 7, 14, 21d
49	Friedman & Bonferroni-corrected Wilcoxon paired signed rank tests	Y	N	AST inc from BS in T/t gp from BS to 2wk followed by dec Inc in CC gp from BS to 1wk followed by dec AST level in comp >tn on 1wk	14d	GCF flow in T/t=CC>AC gp	+ve correl of mechanical stress to AST levels, T/t>CC	sn inc in T/t &CC vs AC gp: 1, 2, 3, 4w sn inc in T/t vs CC gp: 1, 2wk AST level in comp >tn on 1wk
50	One-way ANOVA was used for multiple group and Student t test for group-wise comparisons	Y	N	inc in ALP b/w 21d & 28d :of 200% in active TB gp, of 260% in Rt screw gp	TB: 21d Rt screw:28d	Space closure rate, root resorption, Rt, anchorage loss with Hycon screw were assessed	+ve correl of ALP in Hycon screw gp with actvn of screw	Sign diff in ALP on 21d & 28d b/w TB & Rt screw gp
51	independent t tests, _2 tests, or Mann-Whitney, intraexaminer reliability - concordance correlation coefficient (CCC) & Bland-Altman method	N	Y	MMP8,9, MMP8/TIMP1, MMP9/TIMP1, resistin at BS>1h>1wk>compl of Aln CRP, MPO, TIMP, RANKL inc from BS to compl of Aln Adiponectin BS<1h<1wk>compl of Aln Leptin dec from BS to compl of Aln	NM	resistin at BS>1h>1wk>compl of Aln CRP, RANKL inc from BS to compl of Aln Adiponectin BS<1h<1wk>compl of Aln Leptin dec from BS to compl of Aln	Mediators correl with Aln rate- MPO, RANKL, Leptin, Resistin	MPO at BS<1h<1d<compl of Aln
52	Fisher’s PLSD followed by post hoc, Bonferroni- Dunn	Y	N	ALP on cmp site: 0>2wk>4wk<1y tn site: 0<2wk<4wk<1y	tn site: 1y cmp site: before actvn	NM	+ve correl of intermolar distance with ALP level in tn site	tn site:0 (before actvn) < 4wk, 0<1y cmp site: 0>4wk, 0<1y, 2wk>4wk
53	paired & unpaired ‘t’ test and ANOVA.	Y	N	MPO inc from BS to 2h in HANT, SE, MSSS gp	2h	NM	MPO in HANT>SE>MSSS	sn diff in MPO b/w SE & MSSS :2h, 2wk, b/w HANT & MSSS:2h, b/w SE & MSSS:1wk
54	Chi-square Student’s t-test, and one-way analysis of variance	Y	N	MPO inc from BS to 2h in HANT, SE, MSNiTi gp, HANT>SE & MS NiTi:2h	2h	NM	NM	sn diff b/w SE & MSNiTi: 2h, 1, 2wk, b/w HANT & MSNiTi:2h
55	Independent & paired sample t- test	Y	N	AST inc from BS to 1wk, then dec in Exp gp	1wk	NM	NM	Levels greater in Exp than Cn gp at 1, 2, 3, 4wk

A -article, sts -statistically, al -analysis, ap -applied, cf -confounders, rg -regulation, Pk -peak, sd -secondary, oc -outcome, cr -correlation, sn -significant, Y -yes, N -no, NM -not mentioned, inc -increase, dec -decrease, fluct -fluctuated, h -hour, mon -month, d-day, wk-week, tot -total, prot -protein, conc -concentration, mg -milligram, ml -millilitre, g -gram, > -greater than, VAS -visual analogue scale, C-canine, mov-movement, b/w-between, cn-continuous, &-and, F-force, Asc-associated, gen-genetic, GCF-gingival crevicular fluid, compd-compared, B-baseline, IL -interleukin, ΒG-beta glucoronidase, TNFα-tumour necrosis factor alpha, SD-short duration, LD-long duration, HG-, RDG-, Diff-difference, vol-volume, Rt-retraction, if-inflammation, Avg-average, cyt-cytokine, chemo-chemokine, kwn-known, MOP, PI-plaque index, BOP-bleeding on probing, Exp-experimental, c-control, Avg-average, Mx-maxilla, ct-contralateral, differen-differentiation, se-separator, gp-group, cmp-compression, tn- tension, kPa-kilopascal, max-maximum, gw-growth, T-tooth, Oc-osteoclast, RDG- Rapid canine distalisation group, HG- hybrid reactor group, Rt- retraction, Aa-Actinobacillus, rd-reading, wi-wire, lig-ligature, Ad-adult, RANKL-receptor antagonist nuclear kappa ligand, OPG-osteoprotegerin, IL-1RA-interleukin 1 receptor antagonist, therm-thermoplastic, t-PA-plasminogen, TIMP-Tissue inhibitor metalloproteinase, MPO-myeloperoxidase, ortho-orthodontic, cys-cysteine, TSP-thrombospondin 1, NGAL-neutrophil gelatinase-associated lipocalin, GM-CSF-Granulocyte-macrophage colony-stimulating factor, Niti-nitinol, Cp-Cathepsin, Cys-cysteine, Ix-Index, T-tooth, MMP-matrix metalloproteinase, TRAP-Acid phosphatase, ALP-Alkaline Phosphatase, Ms-mesial, D-distal, si-site, Lvl-level, lig-ligation, ACP-acyl carrier protein, PM-premolar, Mo-molar, CI-central incisor, vol-volume, AST-aspartate transaminase, crw-crowding, minm-minimum, ado-adolescent, rec-rectangular, OTM-orthodontic tooth movement, AL-after loading, TT-test tooth, b/w-between, enz-enzyme, Ix-index, pl-plaque, scr-score, Bac-bacterial, PD- probing depth, PL-supragingival plaque, actv- activity, actvn- activation, compl-completion, reactivation-reactvn, SE-superelastic NiTi, HANT- heat-activated NiTi, MSSS- multistranded stainless steel, vol-volume.

An initial upregulation in enzymes for bone resorption and matrix degradation like TRAP, ACP or MMPs and an immediate decrease in bone formative ALP corresponded with Burstone’s initial phases of OTM. Different MMPs responsible for extracellular matrix (ECM) breakdown are increased at variable times in OTM,^13^ ^,^ ^15^ ^,^ ^17^ ^,^ ^18^ ^,^ ^20^ ^,^ ^22^ ^,^ ^27^ ^,^ ^50^ as early as 1hr or till completion of alignment.^50^ MMP-9 increased in 4hr, peaked at 8hr using stainless steel ligatures for canine retraction in one study, while MMP9/NGAL ratio peaked in 72hr in another study.^13^

MMPs also varied with different magnitudes of force as MMP-9 peaked in 4hr in a study using 100g force for canine retraction,^18^ compared to another study using 150g force in which MMP3, 9 and 13 peaked in 24hr.^20^ The difference in peaks of various MMPs can be explained on the basis of difference in their roles in bone turnover and remodeling with orthodontic forces.^59^ MMP-9 is responsible for cleavage of denatured collagen, i.e gelatin;^60^ MMP-13 dissolves native fibrillar collagen; MMP-1 is an interstitial collagenase hydrolyzing mainly type III collagen,^61^ and MMP-3 is responsible for activation of MMPs 8 and 9.^62^ Hence peaks of MMP8 and MMP9/NGAL ratio at 14d^17^ and 72hr,^13^ respectively, occur subsequent to peak of MMP-3 in 1hr/24hr.^17^ ^,^ ^20^ In vitro studies also support rise in MMPs in orthodontic forces, specifically MMP-1,2 mRNA and protein production in human gingival and pdl fibroblasts^63^ ^,^ ^64^ and MMP-1,2, 9 in gingival tissue of dogs.^60^

On the other hand, no significant change in MMP levels were seen in control teeth where no orthodontic force was applied.^17,22^ This clearly supports MMPs as key mediators of remodeling in OTM.

MMPs are also shown to vary with site (tension and compression) in a time-dependent manner, as supported by in vitro models on pdl fibroblasts.^65,66^ Current review showed an increase in MMP1,2 in 1-3hr on tension site (TS) of maxillary canine after activation of NiTi spring while in compression (CS), MMP1 increased at 1hr and MMP2 later, at 8hr.^22^ MMP-9 also increased from 4hr to 7d on compression site in another study.^13^ This upsurge in levels indicate initial collagen turnover and disintegration of ECM on both tension and compression sites in initial phases of OTM. 

Contrary to the MMPs, CS showed a significant increase in GCF levels of MMP inhibitors, TIMP-1 at 4hr and TIMP-2 after 7d during retraction of canines, coinciding with lag phase where tooth movement slows down.^18^ ^,^ ^50^ At TS, a significant increase in TIMP1 and 2 levels was seen at 4hr, 7d and 42d. This finding is in agreement with the results of a study by Bildt et al^67^ where a continuous force with NiTi spring of 150cN was applied for retraction and an increase in MMP1 and TIMP1 was seen on pooled samples from resorption (corresponding to compression) and apposition side (tension) but no trace of TIMP2 was found. The mechanism of action of TIMP-1 stimulates release of MMP1,^68^ an interstitial collagenase, associated with normal tissue remodeling or stretch of pdl fibers, hydrolysing mainly type III collagen.^64^ Also, TIMP-1 increases in smaller amounts on the site of compression, while retraction due to stimulation of bone resorption but in higher amounts on tension, it decreases bone resorption.^67^ A study by Garlet et al.^69^ provided evidence of greater expression of TIMP-1 mRNA on TS and MMP-1 mRNA on CS and TS of experimental teeth compared with the control.

Besides MMPs, histological studies on rats provide evidence of other enzymes for bone resorption predominant in CS in early phases of OTM followed by bone deposition in TS.^70,71^ In accordance, the current review also shows resorptive enzyme -ACP in initial 3-5d of tooth movement.^14^ Few studies on retraction with continuous forces document an initial rise in ACP both on TS and CS with a peak in 14d^42^ and 21d.^46^ Initial resorption is followed by a late phase of bone deposition (7-14d) marked by an increase in bone formative ALP levels,^37,45^ seen both in TS and CS of alveolar wall. Increase in ALP occurs by increasing the local concentration of phosphate ions after hydrolysis of phosphomonoester bonds, thus bone mineralisation. Highest serum ALP activity in humans has been correlated with greatest osteoblastic activity during growth spurts.^72^ ^,^ ^73^ The current review has 17 studies evaluating ALP in association with type, site and magnitude of force. ALP levels increased at TS in continuous retraction forces by NiTi spring as well as in gradually increasing force from 50 cN to 150cN at 2wk, showing a predisposition towards bone deposition.^9^ A study in rats supported osteoid deposition in the lacunae on TS in 80-120d.^74^ The current review shows peak in ALP levels at 2wk on continuous force application of 150cN, 100g or 150g force^9^ ^,^ ^10^ ^,^ ^14^ ^,^ ^24^ ^,^ ^28^ ^,^ ^45^, with greater levels on TS compared to CS. This is followed by fall in ALP levels corresponding to hyalinised tissue removal and initiation of post lag phase.^9^ ^,^ ^24^ Magnitude of force was another determinant of variation in ALP. Decrease in ALP levels seen at 1hr, 1d after intrusion by TMA spring is believed to be caused by heavy forces leading to a hyalinised zone.^25^ Conversely, distalisation of molars with heavy cF of 250g^31^ showing high ALP levels at both TS and CS and ALP levels greater in 150g than 100g force,^34^ were attributed to extensive osteoblast recruitment on application of heavy forces.^9^ One study showing decreased ALP levels on both TS and CS of canine retraction with push coil spring was probably due to combination of bodily and tipping movement, which precludes pure compression and tension areas.^38^ ALP also varied with type of force: one study compared levels in Hycon^®^ screw with active tie-backs for retraction. A significant difference was seen at 3 and 4 wk of retraction with levels in Hycon screw group 260% higher after one half turn twice weekly activation, compared with 200% increase in active tie-back group.^49^ This may be ascribed to elastomeric force decay to 30-40% of original force in 3 weeks. Another study on maxillary expansion by hyrax followed by retention noticed fall in ALP levels on CS and TS till four weeks of activation, followed by peak at 1yr on TS, thus indicating bone apposition during retention period.^51^

Contrary to ALP, TRAP or ACP facilitates dissolution of bone minerals by forming a highly acidic extracellular environment and are potent osteoclast biomarkers expressed in areas of compression.^74^ The present review supports rise in TRAP levels at CS more than TS to reach peak at 1wk,^33^ 2wk^11^ and 4-5wk.^34^ ^,^ ^36^ This is supported by histochemical study by Casa et al,^75^ suggestive of appearance of mononuclear TRAP positive cells on application of forces at 2wk and multinucleated TRAP positive cells at 3 and 4wk. Even ACP activity was maximum at 3d, followed by its reversal, explained by natal release of enzymes from surface of osteoclasts.^14^ A secondary outcome of faster rate of OTM with minimal lateral and apical root resorption was noticed with higher levels of TRAP in 150g, compared with 100g force.^34^ ^,^ ^36^

The consummation of bone resorption occurs by resolution of organic matrix mediated by lysosomal cysteine protease cathepsin B that is increased 1d after application of 100-150g or 250g retraction force by E chain,^21^ ^,^ ^30^ while levels of inhibitor cystatin decreases in 1d.^21^ In association, plasminogen activator (t-PA) and its inhibitor (PAI) responsible for extravascular fibrinolysis, reach peak at 24hr only to fall later at 7d.^23^

AST is another cytoplasmic enzyme released in extracellular environment after cell membrane lysis following necrosis^76^ and has been evaluated in 10 studies in the current SR. Peak levels of AST were seen at 1wk,^11^ ^,^ ^40^ ^,^ ^41^ ^,^ ^54^ 2wk,^14^ ^,^ ^48^ and 4wk.^28^ ^,^ ^39^ This may be explained on the basis of increase in AST activity for 14d due to hyalinization of pdl in compression zone, decreased later upon resolution of hyalinized area by macrophages.^14^ The formation of hyalinised zone and cellular necrosis may cause higher levels on CS than TS in retraction cases^39^ ^,^ ^48^ and also in 150g force, compared to 100g.^11^ ^,^ ^34^ But, such sporadic evidence could not be definitive for site predilection. Rather this enzyme has been associated more with destruction of gingival tissues in experimental and chronic periodontitis^77^ and subgingival colonization with arch wire ligation^41^ than orthodontic force application. 

The current review has also monitored LDH, an enzyme released from cytoplasm to extracellular space after cell death in gingivitis or periodontitis^78^ as well as in orthodontic treatment.^16^ ^,^ ^26^ Variation in LDH levels were recorded with type, magnitude and direction of application of force. Continuous force of 125g with NiTi spring showed increase in levels at 7d to peak at 14d,^26^ 21d^19^ and 28d,^48^ but remained higher in CS than TS at 1.5 N,^35^ thus favouring its release after cell death. Timing of increase varied with force level, with an early increase seen at 2wk in heavy force of 250g applied for molar distalisation.^26^ compared with rise in 3wk in 125g force.^19^ ^,^ ^47^ However no significant difference in LDH levels could be correlated to high friction between self-ligating brackets and thermoelastic or superelastic Nitinol wires, as the forces produced by frictional resistance are insufficient for LDH release.^16^ One study supporting greater LDH levels in teeth undergoing retraction compared with controls was excluded from this review because of its cross-sectional study design.^79^ It supported LDH as a sensitive marker of the pdl metabolism changes during OTM. 

Other inflammatory mediators like MPO and βG were also evaluated in this review. MPO released from PMNLs (polymorphonuclear leukocytes) is a sensitive marker for inflammation and pain associated to OTM and showed an early increase at 2hr.^8,12,50,52,53^ In cases of alignment, the levels of MPO increase from baseline to 1hr to 1d till completion of alignment, correlating it with inflammation caused by NiTi wire alignment.^50^ Studies on MPO also supported superelastic NiTi wires as best alignment wires, giving low continuous force and rapid tooth movement, showing higher MPO levels at 2hr, compared with heat-activated NiTi or multistranded NiTi or stainless steel wires.^52^ ^,^ ^53^ Studies also mentioned increase in lysosomal enzyme, βG released from PMNLs after 14d of heavy interrupted force for mid-palatal hyrax expansion in adolescents.^7^ ^,^ ^31^ However, the levels remained high till 28d in retention, probably due to elastic recoil of stretched supracrestal gingival fibers.^7^ ^,^ ^31^

The risk of bias assessment in QAI though indicated all studies as moderately or highly sensitive, revealed certain strengths and weaknesses of variable study designs (Table 7). While the objectives of the studies, selection criteria and orthodontic mechanics were generally clear, they strikingly lacked sample size calculation with only one study indicating the same.^9^ The authors took 5 as the sample size for inclusion, based on statistician’s advice. Randomization of experimental teeth/ side / patients falling into study and control group have been clearly stated in only 21 out of 48 studies, suggesting substantial bias in all studies. The present SR deals with biomarker evaluation in GCF, hence the GCF handling characteristics have been adequate in all studies. However, the specification of time, temperature and humidity at the time of GCF collection was a major shortfall, with only four studies mentioning it. The statistical significance of the results, wherever applicable, have been stated in all the studies, but none of the studies mentioned dropouts or confounders, which might influence the results. 

**Table 7:** Results of quality assessment of 48 studies for inclusion of studies in the review

S. No.	Criteria (29)	Response		
		Yes	No	Unclear
I. Study design (18)				
1.	Objective: objective clearly formulated	48	-	-
2.	Sample size: considered adequate	2	-	46
3.	Spectrum of patients representative of patients receiving the test in practice	48	-	-
4.	Ethical clearance mentioned	40	8	-
5.	Selection criteria: clearly described	48	-	-
6.	Randomization: stated	21	27	-
7.	Baseline characteristics: clearly defined	47	1	-
8.	Control: clearly defined	46	-	2
9.	Orthodontic mechanics explained in sufficient detail to permit replication of experiment	45	1	2
10.	Orthodontic force: clearly specified	35	12	1
11.	Description of execution of index test: sufficient to permit replication of test	45	-	3
12	Absence of time difference between index test & control: mentioned	36	12	-
13.	Index test executed at specified time and environmental conditions	4	44	-
14.	Use of proper indices for assessment of gingival & periodontal status (Pre-treatment)	40	8	-
15.	Use of proper indices for assessment of gingival & periodontal status (at each observation time)	17	29	2
16.	Oral hygiene regime-mentioned	32	3	13
17.	Prophylaxis done (Pre-treatment)	34	14	-
18.	Prophylaxis done(at each observation time)	11	37	-
II. Study measurements (3)				
1.	GCF handling characteristics: explained	47	-	1
2.	Measurement method: appropriate to the objective	48	-	-
3.	Reliability-adequate level of agreement	48	-	
III. Statistical analysis (5)				
1.	Dropouts: dropouts included in data analysis	1	47	-
2.	Statistical analysis: appropriate for data	48		-
3.	Confounders: confounders included in analysis	-	48	-
4.	Statistical significance level: P value stated	48	-	-
5.	Confidence intervals provided	48	-	-
IV. Study results and conclusions (3)				
1.	Index test compared to baseline	48		
2.	Index test compared to control	48		
3.	Conclusions: specific	40		8

*Index test: Refers to collection of GCF at each observation interval in treatment teeth.

Despite the various shortcomings noticed in the study designs, the current evidence has generated ample evidence related to enzymes in OTM and has also opened new arena for future research in this direction. 

Perhaps a most exciting area of research will involve biological basis of tooth movement with different ligation modes of brackets. Further studies could be conducted with LDH as marker for high frictional resistance in different combinations of brackets and wires, as only single study in this SR found no significant change in LDH in initial OTM with self-ligating brackets and superelastic or thermoactive archwire. Another split-mouth study correlating biomarker level with microbial colonization in different ligation modes showed a significantly greater level of AST in arch wire ligation than self-ligation, associated with greater microbial count.

An interesting correlation of MPO with pain was established with an early increase in MPO within 2hr of force application, coinciding with initial pain incidence in orthodontic patients. βG has been explored for its association with the most suitable wires for alignment and could be explored further in different types and magnitudes of forces.

Based on similarity between peri-implant fluid (PIMF) and GCF, the mediators studied in GCF could also be evaluated in PICF to assess stability of contemporary orthodontic anchorage devices, micro-implants, as has been suggested by study of interleukin 1β in PIMF.^80^

Despite the heterogeneity in study design and categories of enzymes studied in literature, this SR provides an essential overview of the mechanism by which enzymes play a role in bone apposition, resorption as well as ECM degradation. The current SR also correlates mediator levels in GCF with phases of OTM at different magnitudes and types of forces and also ligation modes. It goes a step further in suggesting the potential areas of research in this field, based on individual studies designed for associations of mediator levels with ideal orthodontic force magnitudes, method of ligation and periodontal status, thus setting a direct implication in clinical practice. 

## CONCLUSIONS

1. Orthodontic force induces change in levels of multiple enzymes detectable in GCF. These are:
  a) cytoplasmic enzymes released in extracellular environment after cell lysis (LDH, AST), b) Inflammatory markers released from PMNs (MPO, βG), c) enzymes involved in bone and tissue remodelling by bone resorption (TRAP, ACP), d) bone apposition (ALP) or dissolution of organic matrix (Cp, Cys, tPA, PAI) and e) various categories of MMPs responsible for degradation of ECM (MMP1, 2, 3, 8, 9, 13).
2. Compression sites showed early increase in levels of MMP1, MMP2, TIMP1, MMP9 between 1-4hr, and late peak in TIMP2, TRAP, AST after 7d, 4-5wk and 8-12wk, respectively.
3. Tension sites showed significant increase in ALP after 7d, MMP1 between 1-3hr and TIMP 1 and 2 levels at 4hr, 7d and 42d.
4. Distinction between TS and CS could be made with levels of TRAP, AST, LDH, MMP9, being greater on CS than TS, and ALP greater on TS.
5. ALP, TRAP levels were greater in 150g force than 100g force. An early rise in AST levels was seen in 150g force at 3 and 4wk, as compared to 100g force at 4 and 5 wk.
6. Mechanical stress with continuous force of NiTi spring causes increase in MMPs 1, 3 in 24hr in CS and of ALP as early as 7d in TS.
7. No significant association between levels of MMP-9 or AST and growth status could be established as adult and adolescents, gave no significant difference in levels.
